# Negative Descriptors of Patients With Sickle Cell Disease in the Electronic Health Record

**DOI:** 10.1001/jamanetworkopen.2026.6458

**Published:** 2026-04-13

**Authors:** Austin Wesevich, Alexandria Vangelatos, Michael Sun, Elizabeth L. Tung, Monica E. Peek

**Affiliations:** 1 Section of Hematology/Oncology, University of Chicago, Chicago, Illinois; 2 Department of Medicine, University of Chicago, Chicago, Illinois; 3 Section of General Internal Medicine, University of Chicago, Chicago, Illinois

## Abstract

**Importance:**

Patients with sickle cell disease (SCD) may experience clinician biases due to intersecting stigmatizing factors because most patients with SCD racially identify as Black, have pain as the most common presenting symptom, and are often treated with opioids.

**Objective:**

To examine intersecting stigmatizing identities of patients with SCD—identifying as Black, presenting with pain, and being treated with opioids—and compare differences in negative descriptors in the electronic health record relative to SCD.

**Design, Setting, and Participants:**

This cross-sectional, single-center study of outpatient, emergency department, and inpatient encounters examined clinician notes from January 1, 2019, to October 1, 2020. Clinician notes were included for patients who racially identified as Black or had diagnoses of SCD, chronic pain, or opioid use disorder (OUD). Notes were also included for a counterfactual group of patients who were not Black and did not have SCD, chronic pain, or OUD. Data were analyzed from November 2024 to February 2026.

**Exposure:**

Patients with SCD were compared with 4 groups, all without SCD: (1) Black patients, (2) patients with chronic pain, (3) patients with OUD, and (4) the counterfactual group.

**Main Outcomes and Measures:**

Using natural language processing and a machine learning model, clinician notes were contextually coded for the application of 7 negative descriptors: *aggressive*, *agitated*, *angry*, *nonadherent*, *noncompliant*, *noncooperative*, and *refuse*.

**Results:**

A total of 39 871 clinician notes across 18 326 patients were analyzed (mean [SD] age, 47 [23] years); 2183 patients were pediatric (12%), 10 243 were female (56%), 11 137 were Black (63%), 5907 were married (32%), and 5884 had Medicaid (32%). Negative descriptors were present in 220 of 1443 notes (15%) among patients with SCD and 124 of 643 notes (19%) among those with all 4 stigmatizing identities. There were higher odds of a negative descriptor in notes about patients with SCD compared with Black patients without SCD (aOR, 2.46; 95% CI, 1.62-3.73), patients with chronic pain without SCD (aOR, 1.96; 95% CI, 1.18-3.27), and patients in the counterfactual group (aOR, 14.26; 95% CI, 5.92-34.36), but there was no difference compared with patients with OUD without SCD (aOR, 0.76; 95% CI, 0.42-1.38).

**Conclusions and Relevance:**

In this cross-sectional study of 18 326 patients, those with SCD had more negative descriptors than Black patients without SCD or those with chronic pain but at a similar level as those with OUD.

## Key Points

**Question:**

Is there an association between intersecting stigmatizing factors (ie, Black race, pain presentation, and opioid treatment) among patients with sickle cell disease (SCD) and negative descriptors in the electronic health record?

**Findings:**

In this cross-sectional study of 18 326 patients, there were higher odds of a negative descriptor in notes about patients with SCD compared with Black patients without SCD (aOR, 2.46; 95% CI, 1.62-3.73) and patients with chronic pain without SCD (aOR, 1.96; 95% CI, 1.18-3.27), but they had similar odds of negative descriptors as patients with opioid use disorder without SCD.

**Meaning:**

These findings suggest that bias toward patients with SCD may be associated more with opioid use than race or chronic pain.

## Teaser

This cross-sectional study evaluates whether intersecting stigmatizing identities—including Black race as well as having sickle cell disease, chronic pain, and/or opioid use disorder—are associated with the presence of negative descriptors in a patient's electronic health record.

## Introduction

Clinician biases regarding race, ethnicity, socioeconomic status, gender, and chronic disease can negatively affect clinician attitudes, influence clinical decision-making, and lead to worse health outcomes.^1,2,3,4,5,6,7,8,9,10^ This is particularly true for patients with sickle cell disease (SCD) because they have several intersecting identities (eg, minoritized race, chronic disease) that have been associated with implicit biases. Multiple studies have documented clinician biases and their impact on health care for patients with SCD.^11,12,13,14,15,16,17,18^ In a study of hypothetical clinical vignettes comparing biased vs neutral language to describe patients with SCD, physician trainees were less aggressive in managing the pain for a patient described with biased language.^15^ These findings underscore the potential for biased language to negatively impact sickle cell care.

Most patients with SCD in the US are Black and are thus at risk for experiencing race-based biases from physicians and other clinical staff when accessing health care. Prior research indicates that at least one-third of Black patients have experienced discrimination in health care.^9,19,20,21,22,23,24,25,26,27^ Patients with SCD may also face disease-related biases given stigmas associated with chronic disease, chronic pain, and opioid use.^28,29^ Health care professionals have biases regarding patients with chronic pain,^30^ and Black patients are disproportionately affected by chronic pain.^31^ The combination of stigmatizing identities likely contributes to clinicians prescribing fewer opioids to Black patients with pain than White patients, a disparity that is further exacerbated when patients have opioid use disorder (OUD).^32,33,34^ Acute vaso-occlusive episodes (VOEs) account for roughly 60% of emergency department visits and 95% of hospitalizations among patients with SCD.^35,36^ These admissions are largely to augment pain management with intravenous opioids.^37,38,39,40^ In addition to acute VOE, patients with SCD experience chronic pain, with many adults with SCD reporting pain most days of the week.^41,42,43,44^ Because opioids are often prescribed to manage chronic pain, patients with SCD likely face bias related to opioid use even if they do not have OUD.^16,45,46,47^

While there is durable evidence of inequitable treatment of patients based on race, chronic pain, and opioid use, there is a paucity of literature about the relative contributions of each of these factors on clinician biases toward patients with SCD.^3,48,49,50,51,52^ In 1 study,^16^ patients with SCD reported higher rates of discrimination attributed to disease rather than race, and reports of racial discrimination were higher than those among the general patient population. It is unclear how the multiple marginalized identities of patients with SCD determine their differential attributions of clinician bias (eg, race, social status, pain), and how accurate these differential attributions may be. This study examined whether there are differences in the use of negative descriptors about patients with SCD in the electronic health record (EHR) compared with those with intersecting stigmatizing identities, including Black race, chronic pain, and OUD.

## Methods

### Data and Sample

This cross-sectional study followed Strengthening the Reporting of Observational Studies in Epidemiology (STROBE) reporting guideline and included patients seen at the University of Chicago between January 1 and October 1, 2020, for an outpatient, emergency department, or inpatient encounter, and who had received a COVID-19 test.^53^ The history and physical notes of these patients spanned from as early as 1 year prior to the first COVID-19 test; thus, notes included in analyses were from January 1, 2019, to October 1, 2020. Because 83% of the original sample had an encounter during the 5-month period of universal testing for COVID-19, this sample was not affected by selection bias associated with symptom-based testing.^53^ These history and physical notes are referred to as clinician notes rather than medical notes because they were written by physicians, nurse practitioners, or physician assistants and do not include other health profession notes (eg, nursing, physical therapy).^54^ Due to the retrospective nature of this study, it was approved with a waiver of consent by the University of Chicago Institutional Review Board.

### Classification of Patient Groups

Patients were included in this analysis if they fit into 1 of 5 groups. The patient group of interest—those with a diagnosis of SCD—was compared with 4 groups without SCD: (1) Black patients; (2) patients with chronic pain; (3) patients with OUD; and (4) non-Black patients without chronic pain or OUD (ie, counterfactual group). If a patient had any instance of an *International Statistical Classification of Diseases and Related Health Problems, Tenth Revision* (*ICD-10*) diagnosis code used to define these groups (eTable 1 in Supplement 1), then they were presumed to have that diagnosis across all encounters.^55^ If the database did not include diagnostic data for a given patient, their notes were excluded.

### Classification of Negative Descriptors

Consistent with our prior study, we used natural language processing and a machine learning model to code notes for the presence of a relevant, in-context instance of negative descriptors. The detailed methodology for selecting negative descriptors is described in previous work.^53^ We began with 15 negative descriptors: *aggressive*, *agitated*, *angry*, *challenging*, *combative*, *confront*, *defensive*, *exaggerate*, *hysterical*, *nonadherent*, *noncompliant*, *noncooperative*, *refuse*, *resist*, and *unpleasant*. The 4 descriptors *defensive*, *exaggerate*, *hysterical*, and *unpleasant* were omitted because they were not found in any of the notes about patients with SCD, and the 4 descriptors *challenging*, *combative*, *confront*, and *resist* were omitted because there were too few instances (less than 5 total notes of patients with SCD) to allow for comparisons. This yielded a final list of 7 negative descriptors.

### Covariates

Covariates from the electronic medical record included age (pediatric vs adult), sex (male vs female), race (Black vs non-Black), marital status (married vs unmarried), primary insurance (private, Medicaid, or Medicare), non–age-adjusted Charlson Comorbidity Index, and clinical setting (inpatient, outpatient, or emergency department). Clinical setting was dichotomized into outpatient vs inpatient or emergency department given the low frequency of emergency department notes. Patients with missing covariate data were omitted from regression models.

### Statistical Analysis

Descriptive statistics summarized the sociodemographic characteristics of the study population and the use of negative descriptors. Because the comparison groups were not mutually exclusive, sociodemographic characteristics were not compared by group. The frequency of negative descriptors in clinician notes for patients with different combinations of stigmatizing factors were tallied.

In the main models, patients with SCD were compared with each of the 4 comparison groups. Models assessed for the binary outcome of having any of the 7 negative descriptors in a note, as well as for each negative descriptor individually. Outcomes were modeled as a function of having SCD relative to the comparison group. These comparisons were made overall as well as after stratifying by adult vs pediatric. In addition to unadjusted models, we constructed adjusted multilevel logistic regression models to account for important patient-level covariates. Multilevel logistic regression models accounted for multiple notes per patient. These models clustered notes within encounters and encounters within patients. Two-level models that clustered notes within patients were used when 3-level models were not feasible. While most covariates were statistically significant, race was omitted from final models because of multicollinearity, as indicated by high variance inflation factors, and because almost all patients with SCD were racially minoritized as Black.

As a sensitivity analysis, we examined negative descriptors among the full study sample as a function of: (1) racially minoritized as Black; (2) chronic pain; and (3) OUD. Using multivariable multilevel logistic regression, we calculated the average marginal effect of each stigmatizing factor on the presence of a negative descriptor. This model included the full study sample rather than only those with SCD given collinearity between SCD and being racially minoritized as Black.

Because 99% of patients with SCD racially identified as Black, additional sensitivity analyses were performed to isolate the impact of disease-related bias. In other words, only Black patients with a stigmatizing diagnosis were compared with Black patients with SCD. First, Black patients with SCD were compared with Black patients with chronic pain but without OUD to isolate chronic pain. Then, Black patients with SCD were compared with Black patients with OUD but without chronic pain to isolate OUD. These comparisons allowed isolation of each factor—chronic pain and OUD—as a single variable of comparison and eliminated race as a covariate. A 2-sided statistically significant threshold of *P* < .05 was used. All analyses were done using Stata version 19.0 (StataCorp). Data were analyzed from November 2024 to February 2026.

## Results

A total of 39 871 clinician notes across 18 326 patients (mean [SD] age, 47 [23] years; 2183 pediatric patients [12%], 10 243 female patients [56%], 11 137 Black patients [63%], 5907 married [32%], and 5884 had Medicaid [32%]) were included in the final sample. Of the clinician notes, 21 364 were from inpatient settings (54%), 17 977 from outpatient settings (45%), and 530 from emergency department (1%) settings (eTable 2 in Supplement 1). The 243 patients with SCD (1443 notes) were mostly female (140 [58%]), Black (240 [99%]), unmarried (233 [96%]), and insured by Medicaid (168 [69%]) (Table 1). All comparison groups had a higher median age than patients with SCD. Patients with OUD were less frequently female (270 [41%]). Patients in the counterfactual group were more commonly married (2929 [54%]) and had private insurance (3190 [58%]). Of the 243 patients with SCD, 111 (46%) had a diagnosis of chronic pain, and 84 (35%) had a diagnosis of OUD.

**Table 1.** Sociodemographic Characteristics of Patients by Group

Sociodemographic characteristic	Sickle cell disease (n = 243)	Black (n = 10 897)	Chronic pain (n = 3724)	Opioid use disorder (n = 655)	Counterfactual (n = 5458)
Age, median (IQR), y	25 (17-35)	50 (28-65)	60 (48-69)	58 (49-65)	54 (31-67)
Median Charlson Comorbidity Index (IQR)	0 (0-0)	0 (0-2)	0 (0-2)	1 (0-3)	0 (0-2)
Age group					
Pediatric, <18 y	63 (26)	1284 (12)	73 (2)	5 (1)	715 (13)
Adult, ≥18 y	180 (74)	9613 (88)	3651 (98)	650 (99)	4743 (87)
Sex					
Female	140 (58)	6350 (58)	2391 (64)	270 (41)	2762 (51)
Male	103 (42)	4547 (42)	1333 (36)	385 (59)	2696 (49)
Race					
Black	240 (99)	10 897 (100)	2569 (71)	542 (83)	0
Non-Black	2 (1)	0	1059 (29)	108 (17)	5458 (100)
Married^a^	10 (6)	2018 (21)	1247 (34)	126 (19)	2929 (62)
Insurance					
Private	35 (14)	2284 (21)	1056 (28)	60 (9)	3190 (58)
Medicaid	168 (69)	4902 (45)	848 (23)	350 (53)	610 (11)
Medicare	40 (16)	3711 (34)	1820 (49)	245 (37)	1658 (30)

^a^ Percentage married was calculated out of those 18 years or older: 180 patients with sickle cell disease, 9613 Black patients, 3651 patients with chronic pain, 650 patients with opioid use disorder, and 4743 patients in the counterfactual group.

### Sickle Cell Disease vs Comparison Groups

Negative descriptors were present in 220 notes (15%) about patients with SCD. The most common negative descriptors were *refuse* (93 [6%]), *noncompliant* (70 [5%]), and *nonadherent* (34 [2%]). Negative descriptors were present in 257 notes (14%) about patients with OUD, 1609 notes (7%) about Black patients, and 641 notes (7%) about patients with chronic pain (all without SCD); 323 notes (3%) about patients in the counterfactual group had a negative descriptor. Black patients with SCD, chronic pain, and OUD had the highest frequency of negative descriptors in notes (124 [19%]) (Figure and eTable 3 in Supplement 1).

**Figure.**
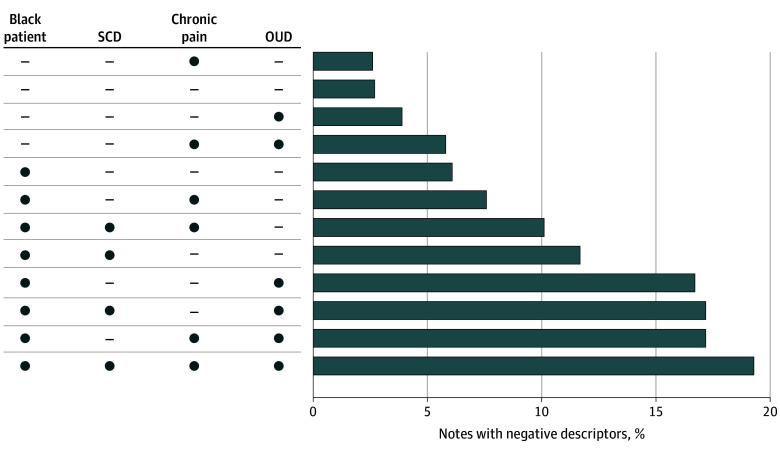
Frequency of Negative Descriptors Across Intersecting Stigmatizing Factors This figure outlines the patients who had 1 or multiple intersecting stigmatizing factors and what proportion of their clinical notes had at least 1 negative descriptor in them. Circles indicate the stigmatizing factor was present and dashes indicate the stigmatizing factor was not present. OUD indicates opioid use disorder; SCD, sickle cell disease.

In unadjusted models, patients with SCD had higher odds of having a negative descriptor in their notes than Black patients without SCD, patients with chronic pain without SCD, and patients in the counterfactual group (Table 2). There was no significant difference in the odds of having a negative descriptor in the notes of patients with SCD compared with the notes of patients with OUD without SCD. This remained true in stratified analyses for adult patients but not for pediatric (eTable 4 in Supplement 1).

**Table 2.** Unadjusted and Multivariable Multilevel Models of Negative Descriptors in Notes About Sickle Cell Disease Relative to Comparators

Negative descriptor	SCD vs Black		SCD vs chronic pain		SCD vs OUD		SCD vs counterfactual	
	OR (95% CI)	aOR (95% CI)^a^	OR (95% CI)	aOR (95% CI)^a^	OR (95% CI)	aOR (95% CI)^a^	OR (95% CI)	aOR (95% CI)^a^
Any negative descriptor	2.31 (1.58-3.37)	2.46 (1.62-3.73)	2.61 (1.73-3.93)	1.96 (1.18-3.27)	0.78 (0.51-1.19)	0.76 (0.42-1.38)	27.7 (12.66-60.51)	14.26 (5.92-34.36)
*Aggressive*	3.18 (0.86-11.74)	1.85 (0.57-6.00)	3.86 (1.14-13.08)	1.11 (0.31-4.03)	1.00 (0.27-3.76)	0.49 (0.06-3.82)	7.02 (0.53-92.25)^b^	2.05 (0.27-15.39)^b^
*Agitated*	0.67 (0.26-1.74)	0.59 (0.22-1.62)	1.34 (0.48-3.75)	1.02 (0.33-3.18)	0.23 (0.10-0.56)	0.23 (0.09-0.60)	1.83 (0.65-5.17)^b^	0.57 (0.12-2.65)
*Angry*	3.88 (1.25-11.99)^b^	3.16 (1.02-9.83)^b^	3.06 (0.91-10.32)^b^	2.19 (0.53-9.04)^b^	3.44 (0.51-22.99)^b^	5.05 (0.33-77.21)^b^	5.94 (1.64-21.58)^b^	6.28 (1.29-30.49)^b^
*Nonadherent*	2.13 (0.95-4.76)	2.42 (1.00-5.87)	2.09 (0.98-4.49)	1.82 (0.62-5.29)	0.54 (0.26-1.11)	0.81 (0.36-1.79)	40.15 (7.41-217.58)	11.73 (2.97-46.38)
*Noncompliant*	2.13 (0.79-5.70)	2.26 (0.79-6.41)	1.80 (0.80-4.06)	1.47 (0.61-3.58)	0.53 (0.12-2.38)	0.61 (0.23-1.60)	39.94 (3.66-435.47)	9.74 (1.57-60.52)
*Noncooperative*	0.87 (0.27-2.78)	0.63 (0.19-2.16)	1.53 (0.40-5.82)	0.58 (0.14-2.34)	0.18 (0.04-0.86)	0.17 (0.03-0.95)	2.81 (0.76-10.34)^b^	1.41 (0.34-5.79)^b^
*Refuse*	2.82 (1.81-4.41)	2.99 (1.91-4.68)	2.90 (1.76-4.79)	2.06 (1.22-3.49)	1.21 (0.71-2.06)	1.39 (0.64-3.01)	18.78 (7.97-44.26)	15.41 (5.18-45.82)

Abbreviations: aOR, adjusted odds ratio; OR, odds ratio; OUD, opioid use disorder; SCD, sickle cell disease.

^a^ Adjusted for sex, age group, marital status, encounter setting, insurance, and Charlson Comorbidity Index.

^b^ Two-level (notes within patients) model output reported rather than 3-level model.

In adjusted models, patients with SCD continued to have higher odds of having a negative descriptor in their notes than Black patients without SCD (aOR, 2.46; 95% CI, 1.62-3.73), patients with chronic pain without SCD (aOR, 1.96; 95% CI, 1.18-3.27), and patients in the counterfactual group (aOR, 14.26; 95% CI, 5.92-34.36). They had similar odds of having a negative descriptor as patients with OUD without SCD (aOR, 0.76; 95% CI, 0.42-1.38). Patients with SCD had higher odds of having the specific negative descriptors *angry*, *nonadherent*, *noncompliant*, and *refuse* than patients in at least 1 of the comparison groups, and patients with SCD had lower odds of having the specific negative descriptors *agitated* and *noncooperative* than patients with OUD without SCD.

### Sensitivity Analyses

The average marginal effect for OUD on a negative descriptor in the EHR was 1.4 times larger than the average marginal effect for being racially minoritized as Black and 4.0 times larger than the average marginal effect for chronic pain after adjusting for covariates (eTable 5 in Supplement 1). In the remaining sensitivity analyses, we omitted non-Black patients and those with both chronic pain and OUD, making the comparison groups smaller than in primary analyses. The 240 Black patients with SCD (1418 notes) were compared with 2349 Black patients with chronic pain without OUD or SCD (5645 notes) and to 322 Black patients with OUD without chronic pain or SCD (664 notes). First, Black patients with SCD had higher odds of having a negative descriptor than Black patients with chronic pain (aOR, 1.97; 95% CI, 1.14-3.42) after adjusting for covariates (Table 3). Second, Black patients with SCD had lower odds of having a negative descriptor than Black patients with OUD (aOR, 0.49; 95% CI, 0.27-0.89) after adjusting for covariates.

**Table 3.** Unadjusted and Multivariable Multilevel Models for Sensitivity Analysis

Negative descriptor	SCD vs chronic pain^a^		SCD vs OUD^b^	
	OR (95% CI)	aOR (95% CI)^c^	OR (95% CI)	aOR (95% CI)^c^
Any negative descriptor	2.40 (1.48-3.90)	1.97 (1.14-3.42)	0.54 (0.28-1.01)	0.49 (0.27-0.89)
Aggressive	5.52 (1.67-18.27)	1.57 (0.42-5.90)	1.76 (0.31-10.00)	1.21 (0.20-7.35)
Agitated	1.89 (0.55-6.52)	1.63 (0.40-6.63)	0.13 (0.05-0.32)	0.12 (0.04-0.34)
Angry	2.81 (0.76-10.41)^d^	1.64 (0.35-7.61)^d^	NA	NA
Nonadherent	2.25 (0.81-6.22)	1.69 (0.53-5.37)	0.38 (0.16-0.91)	0.44 (0.17-1.19)
Noncompliant	1.49 (0.41-5.34)	1.64 (0.37-7.37)	0.50 (0.16-1.61)^d^	0.26 (0.06-1.21)
Noncooperative	1.93 (0.46-8.02)	0.94 (0.21-4.21)	0.11 (0.02-0.64)	0.13 (0.03-0.65)
Refuse	2.58 (1.51-4.44)	2.00 (1.14-3.51)	1.84 (0.90-3.77)	1.43 (0.56-3.65)

Abbreviations: aOR, adjusted odds ratio; NA, not applicable; OR, odds ratio; OUD, opioid use disorder; SCD, sickle cell disease.

^a^ Compared Black patients with SCD to Black patients with chronic pain without OUD.

^b^ Compared Black patients with SCD to Black patients with OUD without chronic pain.

^c^ Adjusted for sex, age group, marital status, encounter setting, insurance, and Charlson Comorbidity Index.

^d^ Two-level (notes within patients) model output reported rather than 3-level model.

## Discussion

While prior work has consistently demonstrated that negative descriptors are more common in the EHR of Black patients,^53,56^ our study uniquely quantifies and compares the odds of documentation with negative descriptors across intersecting and potentially stigmatizing identities of patients with SCD: minoritized race, presentation with pain, and being treated with opioids. In this Chicago-based study of 18 326 patients from an academic medical center, we found that patients with SCD had similar odds of a negative descriptor in the EHR compared with patients with OUD without SCD, 2.0 times higher odds of a negative descriptor in the EHR compared with patients with chronic pain without SCD, 2.5 times higher odds of a negative descriptor in the EHR compared with Black patients without SCD, and 14.3 times higher odds compared with patients in the counterfactual group (ie, non-Black patients without SCD, chronic pain, or OUD). When restricting to Black patients with a single disease-related identity (eg, SCD, chronic pain, or OUD), the findings were similar when comparing patients with SCD to patients with chronic pain, but patients with SCD had lower odds of a negative descriptor than patients with OUD.

To our knowledge, this is the first study to quantitatively measure biased documentation about patients with SCD and potential associations with contributing social identities associated with the disease. OUD appears to be associated with the largest amount of bias, both among patients with SCD and those without SCD. Patients with SCD had similar odds of a negative descriptor in their notes as patients with OUD without SCD. Of note, most patients with SCD in this study did not have a diagnosis of OUD, consistent with multiple studies outlining that OUD is not a significant concern in the general SCD patient population.^57,58,59,60,61,62^ Our findings suggest that SCD amplifies the bias patients experience beyond racial identity and chronic pain, likely due to the conflation of opioid use with OUD.^63^ Research has shown that clinician biases are amplified at the intersection of race and OUD given racial prejudices associated with substance use disorders and negative stereotypes associated with opioid use.^34,47,64,65^ For example, a study found that among patients treated with opioids for chronic pain, Black patients were almost twice as likely as White patients to undergo urine drug testing for illicit drugs and 3-times as likely to have opioids discontinued than White patients if testing positive for cocaine.^66^

The frequency of negative descriptors also varied based on social identity, where patients with SCD and patients with OUD both had approximately 15% of their notes with negative descriptors. Black patients without SCD and patients with chronic pain had about half the prevalence of negative descriptors (7% of their notes). This is in contrast to patients with none of these stigmatizing identities, who had negative descriptors in 3% of notes. Black patients with all 3 stigmatizing diagnoses of interest—SCD, chronic pain, and OUD—had the highest frequency of negative descriptors at 19% of notes. Each stigmatizing factor appeared to be associated with the presence of negative descriptors in clinician notes, with stronger associations with race and OUD than with chronic pain. Taken together, our results suggest that bias against patients with SCD is multidimensional and may accumulate along intersecting stigmatizing identities. Intersectionality is the study of how multiple systems of social stratification (eg, race, gender, chronic disease status) influence an individual’s identity and lived experience, including their emotional and physical health and well-being. Our study corroborates other research about intersectionality and the increased risk of potential harm for persons with multiple marginalized identities, both in society and in health care systems.^67,68,69^

The most frequently used negative descriptors among patients with SCD were *refuse*, *noncompliant*, and *nonadherent*. The negative descriptor with the largest average effect size was *angry*. Prior work has shown that patients with SCD who experience discrimination in health care report lower adherence to physician recommendations.^70^ However, these same patients may then be persistently labeled as *noncompliant*, which could perpetuate discriminatory behaviors from health care workers.^8,71^ Rather than propagate negative labels of patients, clinicians should understand why patients do not want to take a medication or are having trouble with adherence, and then adjust the plan to support patients and document this patient-centered care accordingly. Interventions are needed to break the cycle of confirmation bias and help clinicians better understand the perspective of patients with SCD under their care.^71,72,73,74^ This is also important during transitions of care (eg, prolonged hospitalizations) where negative descriptors in the EHR can impact future treatment by physicians who are less familiar with the patient.^10^

### Limitations

This study has several limitations. First, we used a retrospective design at a single urban academic medical center, which may limit generalizability.^53^ Second, it is possible that patients with SCD are underdiagnosed with chronic pain or OUD. Clinicians may not fully consider or evaluate for the diagnostic criteria of chronic pain or OUD when treating a patient with SCD due to implicit associations between SCD and either pain or opioid use. This may underestimate the prevalence of these comorbid diagnoses for patients with SCD, although it would not change the main models due to the exclusion of SCD among comparison groups. It is also possible that physicians disproportionately apply diagnoses of chronic pain and OUD to patients racially minoritized as Black. If Black patients were more likely to have these comorbid diagnoses, quantification of the association with Black racial identity would be biased toward the null. Third, we indirectly examined the contributions of pain and opioid use to the bias faced by patients with SCD by comparing them to patients without SCD who had either chronic pain or OUD. These are imperfect surrogates as we were limited in our dataset by identifying comparison groups using *ICD-10* codes and there is no *ICD-10* code for chronic opioid use. Thus, the diagnosis of chronic pain served as a proxy for experiencing pain, and the diagnosis of OUD served as a proxy for being treated with opioids. It is possible that patients with SCD would not have had significantly lower odds of a negative descriptor if being compared with those with chronic opioid use rather than those with OUD. Additionally, none of the patients in the comparison groups for the main models had SCD. The experience of pain management for a patient with chronic pain may differ from that of a patient with SCD. It is possible that clinicians respond to pain and prescribe opioids differently for a patient with SCD than a Black patient with chronic pain or OUD who does not have SCD. Fourth, we did not have information on clinicians, such as specialty, race, or ethnicity, that may impact outcomes. Fifth, the comparator groups of Black race, chronic pain, and OUD were not mutually exclusive. Sixth, pediatric analyses were underpowered. Finally, we used *ICD-10* codes at baseline to classify patients into groups for the 22-month study period. Thus, we were unable to account for change in the application of negative descriptors for new diagnoses during the study period.

## Conclusions

In this cross-sectional study of 18 326 patients, we identified the relative strengths of associations of opioid use, minoritized race, and presentation with pain with negative descriptors in notes of patients with and without SCD. The race-based bias that Black patients with SCD experience appeared to be augmented by disease-related biases regarding opioid use and chronic pain. Opioid use was associated with substantially more stigma than chronic pain. Regardless of whether patients had stigmatizing diagnoses or not, Black patients in our study had more frequent negative descriptors than non-Black patients, with the extent of biased language increasing with the number of stigmatizing diagnoses. Antibias interventions must mitigate both race-based and disease-related biases to improve care for patients with SCD and potentially other marginalized patients treated with opioids.

## Notes

Supplement 1. **eTable 1.** Medical Diagnoses of Interest and Corresponding *ICD-10* Codes **eTable 2.** Frequency of Notes and Patients Across Groups **eTable 3.** Notes With Negative Descriptors Across Intersecting Stigmatizing Factors **eTable 4.** Unadjusted Multilevel Models of Negative Descriptors in Notes About Sickle Cell Disease Relative to Comparators After Stratifying by Age Group **eTable 5.** Average Marginal Effects of Stigmatizing Factors

Supplement 2.
Data Sharing Statement
