# Supplementary material for: Negative Descriptors of Patients With Sickle Cell Disease in the Electronic Health Record
Source: JAMA Netw Open. 2026 Apr 13;9(4):e266458. doi: 10.1001/jamanetworkopen.2026.6458 (PMC13077522; doi:10.1001/jamanetworkopen.2026.6458)
Supplement: Supplement 1. — eTable 1. Medical Diagnoses of Interest and Corresponding ICD-10 Codes eTable 2. Frequency of Notes and Patients Across Groups eTable 3. Notes With Negative Descriptors Across Intersecting Stigmatizing Factors eTable 4. Unadjusted Multilevel Models of Negative Descriptors in Notes About Sickle Cell Disease Relative to Comparators After Stratifying by Age Group eTable 5. Average Marginal Effects of Stigmatizing Factors [file jamanetwopen-e266458-s001.pdf]

Supplemental Online Content

Wesevich A, Vangelatos A, Sun M, Tung EL, Peek ME. Negative descriptors of patients with sickle cell disease in the electronic health record. *JAMA Netw Open*. 9(4):e266458. doi:10.1001/jamanetworkopen.2026.6458

eTable 1. Medical Diagnoses of Interest and Corresponding *ICD-10* Codes
eTable 2. Frequency of Notes and Patients Across Groups
eTable 3. Notes With Negative Descriptors Across Intersecting Stigmatizing Factors
eTable 4. Unadjusted Multilevel Models of Negative Descriptors in Notes About Sickle Cell Disease Relative to Comparators After Stratifying by Age Group **eTable 5**. Average Marginal Effects of Stigmatizing Factors

This supplemental material has been provided by the authors to give readers additional information about their work.

eTable 1. Medical Diagnoses of Interest and Corresponding *ICD-10* Codes

Diagnosis or Diagnosis Category	*ICD-10* Codes
Chronic pain	G89.2; G89.4
Opioid use disorders (use, abuse, dependence)	F11
Sickle cell disease	D57.0; D57.1; D57.2; D57.4; D57.8

© 2026 Wesevich A et al. *JAMA Network Open*.

eTable 2. Frequency of Notes and Patients Across Groups

Group	Patients	Notes
Patients with sickle cell disease (SCD)	243	1,443
Black patients without SCD	10,897	22,546
Patients with chronic pain without SCD	3,724	9,404
Patients with opioid use disorder (OUD) without SCD	655	1,777
Non-Black patients without SCD, chronic pain, or OUD	5,458	11,695

© 2026 Wesevich A et al. *JAMA Network Open*.

eTable 3. Notes With Negative Descriptors Across Intersecting Stigmatizing Factors

Characteristic				Total notes	Notes with negative descriptors, No. (%)
Black	Sickle cell disease	Chronic pain	Opioid use disorder		
No	No	Yes	No	2500	70 (2.7)
No	No	No	No	11 695	323 (2.8)
No	No	No	Yes	87	4 (4.6)
No	No	Yes	Yes	270	16 (5.9)
Yes	No	No	No	15 506	943 (6.1)
Yes	No	Yes	No	5645	429 (7.6)
Yes	Yes	Yes	No	298	30 (10.1)
Yes	Yes	No	No	343	40 (11.7)
Yes	No	No	Yes	664	111 (16.7)
Yes	Yes	No	Yes	134	23 (17.2)
Yes	No	Yes	Yes	731	126 (17.2)
Yes	Yes	Yes	Yes	643	124 (19.3)

© 2026 Wesevich A et al. *JAMA Network Open*.

eTable 4. Unadjusted Multilevel Models of Negative Descriptors in Notes about Sickle Cell Disease Relative to Comparators After Stratifying by Age Group

Negative Descriptor	SCD vs Black OR (95% CI)	SCD vs Chronic Pain OR (95% CI)	SCD vs OUD OR (95% CI)	SCD vs Counterfactual OR (95% CI)
Adult Patients				
Any Negative Descriptor	2.42 (1.59-3.69)	2.79 (1.78-4.37)	0.85 (0.55-1.33)	20.7 (9.82-43.7)
Aggressive	5.35 (1.27-22.6)	6.55 (1.75-24.6)	1.53 (0.38-6.18)	13.5[a] (2.29-79.1)
Agitated	0.73 (0.25-2.11)	1.44 (0.49-4.26)	0.25 (0.10-0.65)	2.79[a] (0.81-9.63)
Angry	2.88[a] (0.85-9.78)	2.10[a] (0.59-7.55)	2.23[a] (0.37-13.4)	4.90[a] (1.17-20.5)
Nonadherent	2.66 (1.15-6.16)	2.71 (1.22-6.03)	0.68 (0.33-1.44)	59.8 (8.48-421)
Noncompliant	2.27 (0.80-6.44)	2.33 (0.81-6.70)	0.67 (0.17-2.57)	21.9 (3.53-136)
Noncooperative	1.27 (0.35-4.61)	2.23 (0.52-9.48)	0.24 (0.05-1.21)	5.99[a] (1.69-21.3)
Refuse	2.75 (1.67-4.51)	2.87 (1.66-4.98)	1.23 (0.62-2.44)	14.8 (6.34-34.4)
Pediatric Patients				
Any Negative Descriptor	2.69 (0.95-7.64)	3.32[a] (0.74-15.0)	0.19 (0.01-4.01)	5.23 (1.64-16.7)
Aggressive	-	-	-	-
Agitated	0.36 (0.02-5.34)	-	0.10 (0.00-2.43)	0.46[a] (0.04-5.16)
Angry	-	2.93[a] (0.09-97.5)	-	6.09[a] (0.43-86.6)
Nonadherent	0.39 (0.01-15.0)	1.45 (0.04-57.6)	-	-
Noncompliant	-	1.60 (0.00-875)	-	-
Noncooperative	-	-	-	-
Refuse	4.29 (1.45-12.7)	11.4 (1.54-84.2)	2.05 (0.04-106)	37.0 (1.74-787)

[a]2-level (notes within patients) model output reported rather than 3-level model.

© 2026 Wesevich A et al. *JAMA Network Open*.

eTable 5. Average Marginal Effects of Stigmatizing Factors

Stigmatizing Factor	Unadjusted Multilevel Logistic		Adjusted Multilevel Logistic	
	OR	Marginal Effect	aOR	Marginal Effect
Racially minoritized as Black	4.00	0.048	2.40	0.029
Chronic pain	1.23	0.007	1.37	0.010
OUD	4.65	0.053	3.44	0.041

© 2026 Wesevich A et al. *JAMA Network Open*.
