# Supplementary material for: Negative Descriptors of Patients With Sickle Cell Disease in the Electronic Health Record
Source: JAMA Netw Open. 2026 Apr 13;9(4):e266458. doi: 10.1001/jamanetworkopen.2026.6458 (PMC13077522; doi:10.1001/jamanetworkopen.2026.6458)
Supplement: Supplement 2. — Data Sharing Statement [file jamanetwopen-e266458-s002.pdf]

Data Sharing Statement

Data
Data available: No

Additional Information
Explanation for why data not available: Because of the large amount of patient data that was obtained retrospectively including what was documented in clinician notes, only individual requests for data availability will be considered in consideration of patient privacy.
